# Quantitative Determination and Toxicity Evaluation of Aristolochic Acid Analogues in *Asarum heterotropoides* F. Schmidt (Xixin) and Traditional Chinese Patent Medicines

**DOI:** 10.3389/fphar.2021.761593

**Published:** 2021-11-26

**Authors:** Suyan Liu, Zhong Xian, Yong Zhao, Lianmei Wang, Jingzhuo Tian, Chen Pan, Jiayin Han, Yushi Zhang, Chunying Li, Yan Yi, Chenyue Liu, Dunfang Wang, Jing Meng, Shasha Qin, Fang Wang, Aihua Liang

**Affiliations:** Key Laboratory of Beijing for Identification and Safety Evaluation of Chinese Medicine, State Key Laboratory of Innovative Natural Medicine and TCM Injections, Institute of Chinese Materia Medica, China Academy of Chinese Medical Sciences, Beijing, China

**Keywords:** *Asarum heterotropoides* F. Schmidt, aristolochic acid analogues, content, toxicity, safety

## Abstract

*Asarum* (Xixin), which contains analogues of aristolochic acid (AA), is the only species of the genus *Aristolochia* included in the Chinese Pharmacopoeia 2020. However, the contents and nephrotoxic effects of AA analogs in *Asarum* (Xixin) and its formulations have not been clarified. An automatic, effective solid phase extraction process and UPLC-MS/MS method were established for the pretreatment and quantitative detection of AA analogues in commercially available traditional Chinese patent medicines. The cytotoxicity and DNA damage induced by five analogues of AA were evaluated by CCK8 using human kidney cells (HK-2) and comet assays. HPLC was used to detect the analogues of AA in *Asarum heterotropoides* F. Schmidt (Xixin). The results showed that the contents of AA I, AA II, and AA IIIa were below the detection limit, while AA IVa and AL I presented relatively high contents of *Asarum heterotropoides* F. Schmidt (Xixin), within the range of 66.50–121.03 μg/g and 19.73–43.75 μg/g, respectively. The levels of AA analogues were in the nanogram-per-gram level in the main traditional Chinese patent medicines. AA I and AL I exhibited relatively high cytotoxicity at 48 h in CCK8 assays, while AA II, AA IIIa, and AA IVa showed weak cytotoxicity even at 800–1,000 μM. AA I induced significant pathological alterations and direct DNA damage at 40 mg/kg and 20 mg/kg, respectively. No distinct nephrotoxicity or hepatotoxicity was observed in mice treated with AA II, AA IIIa, AA IVa, or AL I at 40 mg/kg in this study. Consumption of *Asarum heterotropoides* F. Schmidt (Xixin) with controlled doses and periods is relatively safe as the contents of AA analogues in *Asarum heterotropoides* F. Schmidt (Xixin) and its formulations were far below those causing acute toxicity in this study. But, the long-term toxicity of *Asarum heterotropoides* F. Schmidt (Xixin) still needs further study.

## Introduction

Aristolochic acid (AA) exists mainly in plants of the Aristolochiaceae family that have been used in pharmaceutical formulations for hundreds of years to treat various diseases (Heinrich et al., 2009; Li et al., 2017)*.* Exposure to AA is associated with nephropathy and upper tract urothelial carcinoma (UUC) (Heinrich et al., 2009; Wu and Wang, 2013; Yang et al., 2014). AA is also the causative agent of Balkan Endemic Nephropathy (BEN), a chronic, progressive renal disease, which arises from the consumption of bread in which wheat grain is contaminated with the weed of *Aristolochia clematitis* (Jelaković et al., 2012; Jelaković et al., 2019). There are various analogues of AA (AAA) in plants of the Aristolochiaceae family (Kuo et al., 2012; Mao et al., 2017). Among these, AA I and AA II are considered the main active components of *Aristolochia* species (Debelle et al., 2008). AA I and its metabolites can react with DNA to form covalent adducts, such as dA-AL I and dG-AL I, which are characteristic markers of exposure to AAs (Stiborová et al., 2017; Sidorenko, 2020). The adducts can induce adenine-to-thymine (A>T) transversions, which have been detected both *in vitro* and *in vivo* (Chen et al., 2012; Chen et al., 2013).

In 2002, AA I was listed as class I carcinogen by WHO due to its nephrotoxicity and carcinogenicity. Subsequently, most *Aristolochia* plants have been prohibited for clinical use in many countries and districts except *Asarum* (Xixin), as it contains relatively low levels of AA I and AA II (Jong et al., 2003). Furthermore, some studies have shown that most *Asarum* (Xixin) products are not cytotoxic in HK-2 cells *in vitro* (Li Y.-L. et al., 2010; Michl et al., 2017). Recently, traditional Chinese herbal remedies containing AA have been statistically associated with human liver cancer, based on an AA-associated mutation in patients with hepatocellular carcinoma (HCC) patients (Hsieh et al., 2008; Hoang et al., 2013; Ng et al., 2017). The formation of aristolactam–DNA adducts induced significant A>T transversions in *TP-53* and *JAK1* genes, and initiated liver cancer both in mouse and human liver cancer models (Lu et al., 2020). AA I can also promote the invasion and migration of HCC by activating the complement system C3a/C3aR (Li et al., 2020). Although there is still controversy about the role of AA in HCC, its toxicity should not be ignored. As the only species of the genus *Aristolochia* allowed in the Chinese Pharmacopoeia 2020, the rationality for the clinical application of *Asarum* (Xixin) has aroused public concern.

According to our data, there are almost 200 types of traditional Chinese medicines containing *Asarum* (Xixin) on the Chinese mainland. Since AA I can induce an exceptionally long-term persistence of DNA adducts and irreversible nephrotoxicity, the content of AA I should be strictly controlled (Li et al., 2011; Yang L. et al., 2012; Schmeiser et al., 2014). However, the exact contents of AA I and AA II in *Asarum* (Xixin) from natural sources and its formulated products are not clear. Besides the trace amounts of AA I and AA II, there are many other AA analogues in *Asarum* (Xixin) (Wen et al., 2014; Michl et al., 2017). Whether they played a role in the AAN progression was not well known. The toxicities of these analogues of AA may have been neglected (Michl et al., 2014).

One aim of this study was to quantitatively detect the contents of AA I, AA II, AA IIIa, AA IVa, and AL I in *Asarum heterotropoides* F. Schmidt (Xixin) and its formulated products. The other was to assess their *in vitro* and *in vivo* toxicities, to provide a rationale for the clinical application of *Asarum heterotropoides* F. Schmidt (Xixin). HPLC was usually used for the detection and quantification of AA analogs in crude drugs (Li W. et al., 2004; Xu et al., 2013). However, it is not sensitive enough to detect the trace amounts of AA analogues in traditional Chinese patent medicines (Chen et al., 2020). In this study, an efficient solid phase extraction (SPE) and UPLC-MS/MS method was developed to quantitatively detect the contents of AA I, AA II, AA IIIa, AA IVa, and AL I in *Asarum heterotropoides* F. Schmidt (Xixin) of different origins and commercially available traditional Chinese patent medicines. Cytotoxicity, acute toxicity, and comet assays were performed to compare the *in vitro* and *in vivo* toxicity of different AA analogues, to provide a theoretical basis for the safe clinical application of *Asarum heterotropoides* F. Schmidt and its formulated products.

## Materials and Methods

### Samples and Solvents

Fifteen batches of *Asarum heterotropoides* F. Schmidt (Xixin) samples were purchased from different districts of the Chinese mainland with batch numbers XX01–XX15. All were authenticated as the roots and rhizomes of *Asarum heterotropoides* F. Schmidt (Xixin) by Prof. Jinda Hao, Institute of Chinese Materia Medica, China Academy of Chinese Medical Sciences. Traditional Chinese patent medicines containing herbs from the *Aristolochiaceae* family were collected from online and offline pharmacies.

The AA I reference standard (purity >98%) was purchased from the National Institutes of Food and Drug Control (Beijing, China). AA II, AA IIIa, AA IVa, and AL I (purities >98%) reference standards were obtained from Chengdu Push Bio-technology Co., Ltd. (Chengdu, China). Methanol, acetonitrile, formic acid, and ammonium acetate (HPLC and MS/MS grade) were purchased from Fisher Scientific (MA, United States). Aspartate amino transferase (AST), alanine amino transferase (ALT), alkaline phosphatase activities (ALP), creatinine (CREA), blood urea nitrogen (BUN), total bile acid (TBA), and total bilirubin (TBIL) were purchased from Hua Sin Science Co., Ltd (Guangzhou, China).

### Chromatographic Separation and ESI-MS/MS

The Agilent Eclipse XDB C18 column (4.6 × 150 mm, 5 μm) was used for chromatographic separations of the analogues of AA in *Asarum heterotropoides* F. Schmidt (Xixin). The mobile phases were acetonitrile containing 0.1% formic acid (A) and water containing 0.1% formic acid (B). Separations were performed as follows: 0–5 min, 30–30% A; 5–5.01 min, 30–36% A; 5.01–26 min, 36–43% A; 26–28 min, 43–43% A; 28–28.01 min, 43–30% A; 28.01–30 min, 30–30% A. The flow rate was 1.0 ml/min. AA analogues were detected at a wavelength of 254 nm. The column temperature was maintained at 20–23°C. Samples (10 μl) were injected into the HPLC system.

Quantitative determination of analogues of AA in traditional Chinese patent medicines was performed with a UPLC-MS/MS system consisting of an ACQUITY UPLC I-Class coupled to a Xevo-TQS detector (Waters, United States). Chromatographic separation was achieved using an ACQUITY UPLC BEH C18 column (2.1 × 50 mm, 1.7 μm; Waters, United States) with a flow rate of 0.3 ml/min at 35°C. Separation was obtained with the following gradient: 0–1.0 min, 10% B; 1.0–7.0 min, 10–75% B; 7.0–7.2 min, 75–95% B; 7.2–10.2 min, 95% B; 10.2–10.3 min, 95–10% B, 10.3–12.0 min, 10% B (A: water with 0.01% formic acid, B: methanol with 0.01% formic acid). Both mobile phases were added with 5 mM ammonium acetate and treated under ultrasonication for 5 min before usage. The injection volume was 1.0 μl.

Multiple reaction monitoring mode (MRM) and positive electrospray ionization were applied for the quantitative determination. The instrument parameters were as follows: source temperature, 150°C; capillary voltage, 3.5 kV; desolvation gas flow, 800 L/h; cone gas flow, 150 L/h; nebulizer gas, 7.0 bar; desolvation temperature, 550°C. Details of the parent/daughter ions, cone voltages, and collision energies for AA I, AA II, AA IIIa, AA IVa, and AL I are listed in Supplementary Table S2.

### Sample Preparation

*Asarum heterotropoides* F. Schmidt (Xixin) samples were crushed into a powder and 1.0 g was placed in a conical flask, followed by cold immersion with 20 ml of 70% ethanol for 1 h and ultrasonic extraction (250 W, 40 kHz) for 30 min. The supernatant was collected and the residue was extracted again according to the same procedures. Then the supernatants were combined and filtered through a 0.22-μm membrane prior to HPLC determination.

Traditional Chinese patent medicines (including capsules, granules, tablets, powders and water pills) were crushed and filtered through a 60-mesh sieve. Honeyed pill was cut into small pieces. Each 50.0 mg sample was accurately weighed and placed in a 10 ml centrifuge tube, to which 4 ml of methanol was added. The mixture was ultrasonicated for 90 min at room temperature and then centrifuged at 4,000 rpm for 15 min. The supernatant was collected and dried with nitrogen at 45°C. The residue was redissolved with 1.0 ml of 30% methanol and the pH was adjusted to 8.0–9.0 using aqueous ammonia. Biotage Extrahera (Biotage, Sweden) was applied for the automatic solid phase extraction process. Waters Oasis MAX Cartridge 3 cc/60 mg columns (Waters, United States) were first activated by 3.0 ml of methanol and 3.0 ml of water. Then the columns were balanced with 3.0 ml of 30% methanol. The samples were loaded and washed sequentially with 5% aqueous ammonia and 60% methanol, followed by elution with 3.0 ml of methanol and 4.0 ml of methanol containing 8% FA. The eluent was collected and dried with nitrogen. Then the residue was redissolved with 1.0 ml methanol and centrifuged at 13,000×*g* for 15 min before UPLC-MS/MS detection.

### *In Vitro* Toxicity Assays

Cytotoxicity analysis was determined using the Cell Counting kit 8 (CCK8) (Bioss, China). HK-2 was cultured in DMEM/F12 (containing 10% fetal bovine serum, 100 U/ml penicillin, and 100 μg/ml streptomycin; Gibco, Invitrogen) at 37°C in 5% carbon dioxide incubator. The cells were maintained at 80% confluency and the medium was replaced every 3 days for routine cultivation. HK-2 cells (8,000 cells/well) were seeded in 96-well plates overnight. The cells were then treated with analogues of AA for 24 or 48 h at different concentrations (6.8, 27.0, 54.0, 108.0, 216.0, and 432.0 μM for AA I; 12.5, 25.0, 100.0, 200.0, 400.0, and 800.0 μM for AA II; 15.6, 62.5, 125.0, 250.0, 500.0, and 1000.0 μM for AA IIIa; 15.6, 31.3, 62.5, 125.0, 500.0, and 1,000.0 μM for AA IVa; 8.0, 16.0, 32.0, 64.0, 128.0, and 256.0 μM for AL I). Subsequently, the cells were incubated with 10% CCK8 solution for another 2 h. The final concentration of DMSO in the medium did not exceed 1.0% v/v. The absorbance (OD) of each well was measured with a spectrophotometer (Spark; Tecan, Switzerland) at 450 nm and the IC_50_ values were obtained by fitting the curve through nonlinear regression with GraphPad Prism 7.0 (GraphPad Software, CA, United States).

### *In Vivo* Toxicity Assays

The animal experiments were approved by the Research Ethics Committee of the Institute of Chinese Materia Medica, China Academy of Chinese Medical Sciences (ICMM, CACMS). The experiment was carried out according to ethical guidelines and regulations for the use of laboratory animals. A total of 160 ICR mice were randomly divided into 16 groups, each comprising 10 mice (5 males and 5 females). The drugs were administered in a single dose by oral gavage and the dose volume was 0.2 ml/10 g. The control group was treated with 0.5% CMC-Na. Other groups received AA I, AA II, AA IIIa, AA IVa, or AL I, at concentrations of 10, 20, and 40 mg/kg, respectively. Animal weights were recorded every two days. The animals were fasted overnight before experiments and sacrificed on day 14. Blood samples were collected and centrifuged for 15 min at 3,500×*g*. AST, ALT, ALP, CREA, BUN, TBA, and TBIL levels in plasma were examined. SPSS 17.0 (IBM Corp., United States) was used for the statistical and significance analyses. The renal and liver tissues were embedded and stained with H&E. Histological images were obtained through Olympus BX63 (Olympus Corporation, Japan) at a magnification of ×200.

### Comet Assays

ICR mice were separated into seven groups (*n* = 5 for each group) and administrated with saline, EMS (577.6 mg/kg), AA I (20 mg/kg), AA II (20 mg/kg), AA IIIa (20 mg/kg), AA IVa (20 mg/kg), or AL I (20 mg/kg) for three consecutive days, respectively. After anesthetization and perfusion, renal and liver tissues were obtained. Comet analysis was performed 2–6 h after the last lavage and the processes were according to Günter Speit (Speit and Rothfuss, 2012). The Comet Assay software project (CASP) (V1.2.3) was used to analyze the fraction of tail DNA and the Olive Tail Moment. A total of one hundred cells from kidney or liver tissues of each animal were analyzed. The mean, standard deviation (SD) of tail DNA%, and the median of olive tail moment were calculated. Results were analyzed using SPSS 17.0 by one-way analysis of variance (ANOVA) followed by the least significance difference (LSD) test. The significant differences between the groups were tested and defined by *p*-value < 0.05.

## Results

### Quantitative Determination of Analogues of AA in *Asarum heterotropoides* F. Schmidt (Xixin) and Traditional Chinese Patent Medicines

**FIGURE 1:**
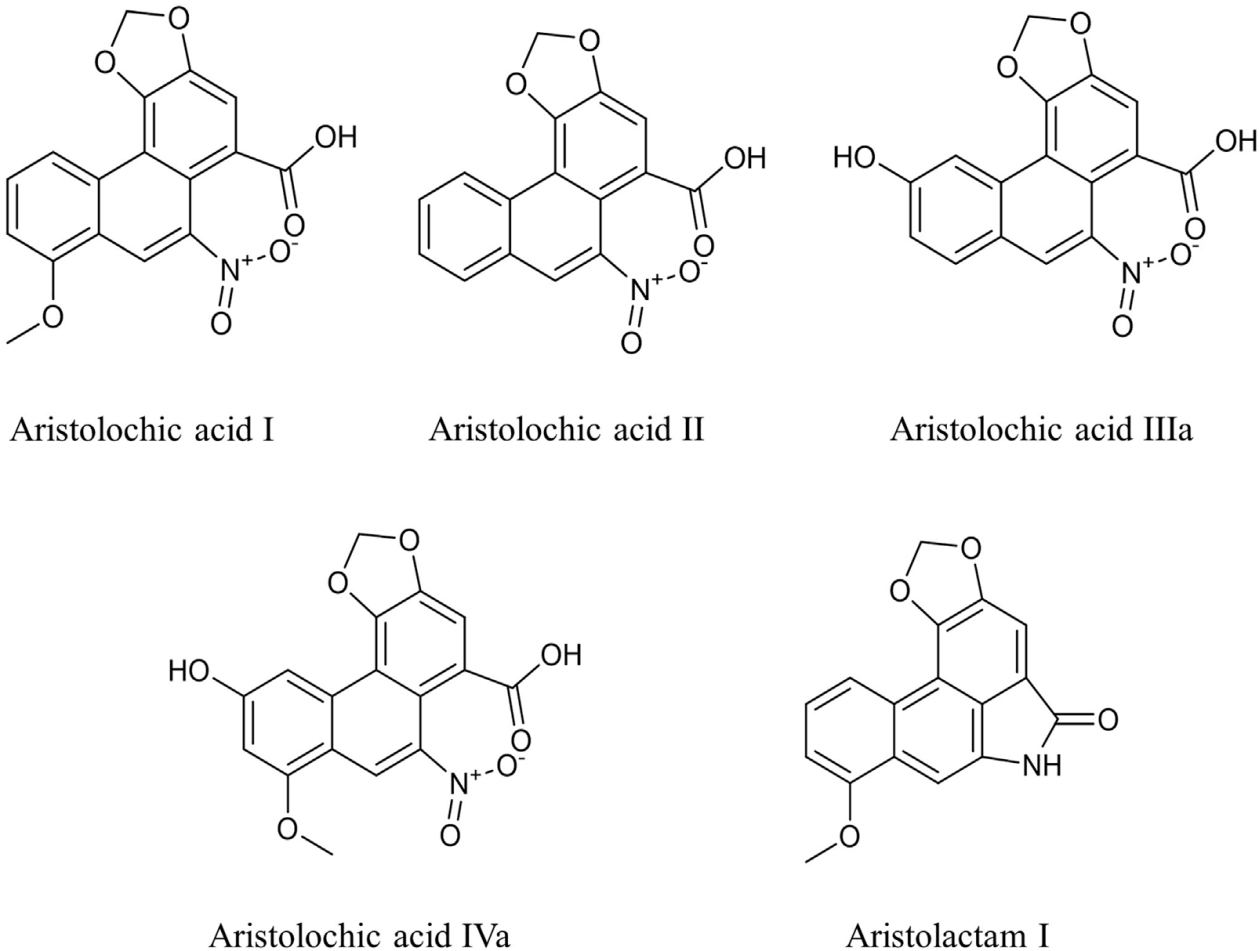
Chemical structures of aristolochic acid analogues in this study.

High-performance liquid chromatography (HPLC) was applied to determine the content of AA analogues (Figure 1) in roots and rhizomes of 15 batches of *Asarum heterotropoides* F. Schmidt (Xixin). The method showed good separation and reproducibility. AA I, AA II, AA IIIa, IVa, and AL I were eluted at 24.36, 20.81, 9.36, 11.23 and 20.31 min, respectively. The quantitative results are shown in Table 1. AA I, AA II, and AA IIIa were not detected in any of the samples, which may be partially attributed to the detection limit of the HPLC instrument. Relative levels of AA IVa and AL I were found in all samples. The contents of AA IVa and AL I were in the ranges of 66.50–121.03 μg/g and 19.73–43.75 μg/g, respectively. XX04 from Liaoning contained the least AA analogues. There were some regional differences in the contents of AA IVa and AL I, but the changes were within the range of 2 fold.

**TABLE 1:** Plant information and contents of AA IVa and AL I in *Asarum heterotropoides* F. Schmidt (Xixin)

No	Species	Origins	Contents (μg/g)	
			AA IVa	AL I
XX01	*Asarum heterotropoides* F. Schmidt	Shanxi	100.15 ± 3.24	25.15 ± 1.06
XX02	*Asarum heterotropoides* F. Schmidt	Jilin	102.23 ± 1.29	25.23 ± 0.35
XX03	*Asarum heterotropoides* F. Schmidt	Liaoning	97.67 ± 3.32	26.49 ± 0.87
XX04	*Asarum heterotropoides* F. Schmidt	Liaoning	66.50 ± 2.31	19.73 ± 2.12
XX05	*Asarum heterotropoides* F. Schmidt	Liaoning	111.62 ± 2.47	30.40 ± 0.99
XX06	*Asarum heterotropoides* F. Schmidt	Liaoning	110.06 ± 1.84	21.45 ± 3.01
XX07	*Asarum heterotropoides* F. Schmidt	Liaoning	109.67 ± 1.99	27.24 ± 0.52
XX08	*Asarum heterotropoides* F. Schmidt	Liaoning	97.02 ± 0.44	25.11 ± 2.98
XX09	*Asarum heterotropoides* F. Schmidt	Liaoning	121.03 ± 1.63	20.62 ± 2.04
XX10	*Asarum heterotropoides* F. Schmidt	Liaoning	109.27 ± 3.10	21.67 ± 0.94
XX11	*Asarum heterotropoides* F. Schmidt	Liaoning	92.24 ± 4.36	24.52 ± 3.97
XX12	*Asarum heterotropoides* F. Schmidt	Liaoning	102.96 ± 0.22	38.08 ± 0.75
XX13	*Asarum heterotropoides* F. Schmidt	Liaoning	96.07 ± 2.71	28.45 ± 1.56
XX14	*Asarum heterotropoides* F. Schmidt	Liaoning	114.85 ± 1.83	43.75 ± 2.13
XX15	*Asarum heterotropoides* F. Schmidt	Liaoning	114.71 ± 3.02	22.59 ± 2.16

Data are shown as mean ± SD. Each content was obtained by evaluating the same sample in triplicate.

An effective and automatic SPE method was established and successfully applied in the pretreatment of traditional Chinese patent medicines. Recovery of each analyte was within the range of 86.50–111.88% at three concentration levels (Supplementary Table S4). The contents of AA analogues (AA I, AA II, AA IIIa, AA IVa, and AL I) in 44 commercially available traditional Chinese patent medicines were obtained by UPLC-MS/MS. AA I, AA IVa, and AL I were detected in most traditional Chinese patent medicines evaluated, while AA II and AA IIIa were only detected in few traditional Chinese patent medicines (Table 2, Supplementary Table S5). 70% of the samples tested contained AA I, with concentrations ranging from 0.010–52.450 μg/g. High levels of AA I (3.41–52.45 μg/g) were found in the Ershiwuweisongshi pill, the Duzhongzhuanggu capsule, and the Jingzhikesoutanchuan pill, which contained processed *Aristolochia debilis* Siebold & Zucc. (Madouling) or *Aristolochia mollissima* Hance (Xungufeng), which have been reported to have a high content of AA I (Yu et al., 2016; Mao et al., 2017). As for medicines containing only *Asarum heterotropoides* F. Schmidt (Xixin), a species of the genus *Aristolochia*, the content of AA I was below 100 ng/g. AA IVa was identified in 86.6% of the samples and the concentrations were within the range of 0.005–1.287 μg/g. Variable content of AL I was detected in all traditional Chinese patent medicines, except for the Shenmei Yangwei and the Shenqi Jianwei Granules. The highest concentration of AL I was found in the Shaqi pill at 5.565 μg/g.

**TABLE 2:** Quantitative determination of aristolochic acid analogues in traditional Chinese patent medicines containing *Asarum heterotropoides* F. Schmidt (Xixin)

Product number	TCM containing *Asarum* (Xixin)	Form	Contents (μg/g)^a^				
			AA I	AA II	AA IIIa	AA IVa	AL I
1	Renshenzaizao	Honeyed pill	ND	ND	ND	0.073 ± 0.003	0.130 ± 0.005
2	Pingganshuluo	Honeyed pill	0.010 ± 0.001	ND	ND	0.128 ± 0.002	0.198 ± 0.005
3	Wumei	Honeyed pill	0.020 ± 0.000	ND	ND	0.249 ± 0.015	0.470 ± 0.063
4	Lusika	Honeyed pill	NQ	ND	ND	0.133 ± 0.008	0.319 ± 0.005
5	Tongrendahuoluo	Honeyed pill	NQ	ND	ND	0.060 ± 0.003	0.076 ± 0.005
6	Ertongqingfei	Honeyed pill	NQ	ND	ND	0.031 ± 0.004	0.205 ± 0.005
7	Sanfenghuoluo	Honeyed pill	NQ	ND	ND	0.089 ± 0.006	0.362 ± 0.016
8	Shiyiweishenqi	Tablet	NQ	ND	ND	0.141 ± 0.004	0.383 ± 0.007
9	Fengshiantai	Tablet	0.026 ± 0.002	ND	ND	0.271 ± 0.020	0.621 ± 0.048
10	Shensanqishangyao	Tablet	0.020 ± 0.001	0.016 ± 0.001	ND	0.023 ± 0.002	0.034 ± 0.001
11	Biyanling	Tablet	0.014 ± 0.008	ND	ND	0.295 ± 0.007	0.153 ± 0.005
12	Houzaoniuhuang	Powder	0.047 ± 0.006	ND	ND	0.731 ± 0.024	1.172 ± 0.094
13	Zhubeidingchuan	Watered pill	ND	0.009 ± 0.001	NQ	ND	0.081 ± 0.006
14	Zhengtian	Watered pill	0.038 ± 0.001	ND	ND	0.634 ± 0.020	2.668 ± 0.051
15	Shaqi	Watered pill	0.096 ± 0.013	ND	ND	0.745 ± 0.023	5.565 ± 0.124
16	Zhuifengtougu	Watered pill	0.021 ± 0.004	ND	ND	0.421 ± 0.006	1.154 ± 0.054
17	Xiaoqinglong	Oral liquid	ND	NQ	ND	0.039 ± 0.007	0.013 ± 0.001
18	Tongtian	Oral liquid	ND	ND	ND	0.118 ± 0.007	0.197 ± 0.004
19	Biyuanshu	Oral liquid	0.014 ± 0.000	ND	ND	0.095 ± 0.010	0.734 ± 0.015
20	Ertongqingfei	Oral liquid	ND	ND	ND	NQ	0.099 ± 0.002
21	Jiuweiqianghuo	Granule	ND	ND	ND	ND	0.065 ± 0.001
22	Chitongxiaoyanling	Granule	0.010 ± 0.00	ND	ND	0.133 ± 0.003	0.088 ± 0.004
23	Xinqin	Granule	ND	ND	ND	0.091 ± 0.004	0.312 ± 0.005
24	Yangxueqingnao	Granule	ND	ND	ND	0.121 ± 0.008	0.189 ± 0.003
25	Xiaoqinglong	Granule	NQ	ND	ND	0.011 ± 0.001	0.035 ± 0.002
26	Weiyanning	Granule	NQ	ND	NQ	0.155 ± 0.003	0.220 ± 0.009
27	Shenqishiyiwei	Granule	ND	ND	ND	0.031 ± 0.004	0.419 ± 0.013
28	Zhennaoning	Capsule	0.031 ± 0.006	ND	ND	0.395 ± 0.008	2.585 ± 0.131
29	Ganteling	Capsule	0.080 ± 0.009	ND	ND	0.045 ± 0.003	1.782 ± 0.122
30	Xinfangbiyan	Capsule	0.017 ± 0.001	ND	NQ	0.076 ± 0.006	1.519 ± 0.067
31	Shiyiweishenqi	Capsule	0.016 ± 0.003	ND	ND	0.204 ± 0.006	0.998 ± 0.037
32	Xingnaozaizao	Capsule	ND	NQ	ND	0.013 ± 0.003	0.636 ± 0.022
33	Shensanqishangyao	Capsule	ND	ND	ND	0.010 ± 0.001	0.055 ± 0.002

a μg/g means μg (AAAs)/g (traditional Chinese patent medicines).

All the data are shown as mean ± SD, and the mean value was obtained by averaging four parallel samples. ND, not detected; NQ, detected, but below the limit of quantitation.

Different dosage forms of traditional Chinese patent medicines with the same composition contained different amounts of AA analogues. For example, the contents of AA I, AA II, AA IVa and AL I in the Shensanqishangyao tablet were 0.020, 0.016, 0.023, and 0.034 µg/g, respectively. However, AA I or AA II were not detected in the Shensanqishangyao capsule. And the contents of AA IVa and AL I were 0.010 and 0.055 µg/g, respectively. The origins of the herb and the processing method of *Asarum heterotropoides* F. Schmidt (Xixin) may account for the content differences of the AA analogues.

### *In Vitro* and *In Vivo* Toxicity of Analogues of AA

**FIGURE 2:**
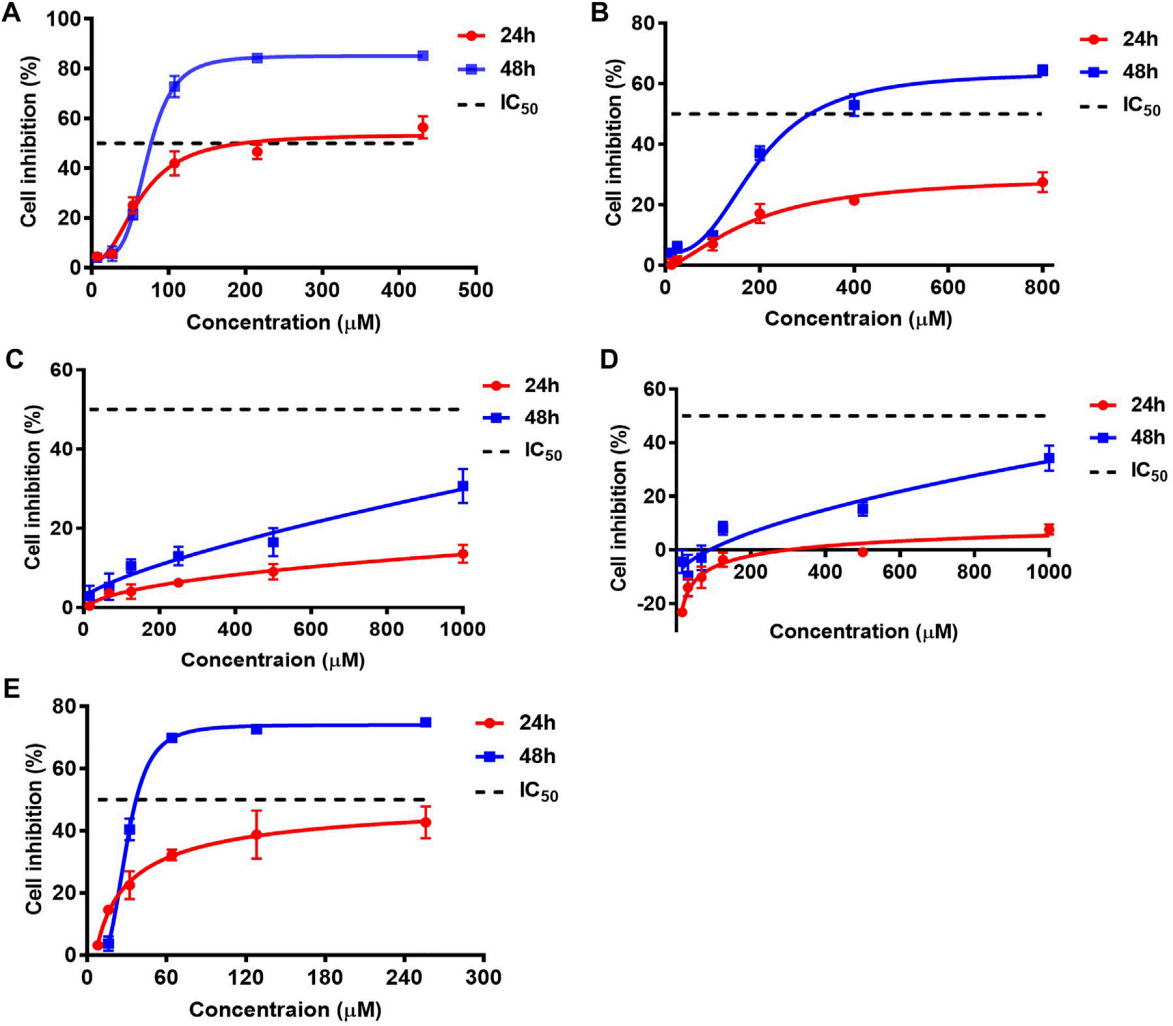
Cell inhibitions of HK-2 after treating with AA I **(A)**, AA II **(B)**, AA IIIa **(C)**, AA IVa **(D)**, and AL I **(E)** for 24 or 48 h. IC_50_ values of AAAs were obtained by nonlinear regression and least square fitting of the data to [inhibitor] vs. response-variable slope (four parameters) model using GraphPad Prism 7.0. Inhibition data were calculated from five parallels at each concentration and repeated for three times.

According to the quantitative results, the contents of AA I, AA II, and AA IIIa were low, while the levels of AA IVa and AL I were relatively high in most *Asarum heterotropoides* F. Schmidt (Xixin)–containing medicines. We questioned whether AA IVa and AL I could play a role in the nephrotoxicity of *Aristolochia*. Thus, we conducted both *in vitro* and *in vivo* toxicity assays. The cytotoxicity of the AA analogues was evaluated using the CCK-8 assay. HK-2 cells were treated with AA analogues for 24 or 48 h. AA analogues showed variable degrees of cytotoxic effects in HK-2 cells, with AA I and AL I exhibiting the strongest cytotoxicity (Figure 2). The IC_50_ values of AA I were 197.3 μM for 24 h and 76.7 μM for 48 h, respectively. AA II showed relatively weak cytotoxicity with IC_50_ > 800 μM for 24 h and 306.5 μM for 48 h. The cell viability was not lower than 50% of control at a concentration of 1,000 μM for AA IIIa and AA IVa at 24 or 48 h, suggesting low cytotoxicity in HK-2 cells. AL I exhibited relatively low cytotoxicity at 24 h with IC_50_ >256 μM. However, the toxicity rapidly enhanced with increasing incubation time. After treatment for 48 h, the IC_50_ value of AL I was reduced to 37.1 μM, exhibiting even stronger cytotoxicity than AA I. Despite the varying cytotoxicity, all AA analogues showed a time- and concentration-dependent inhibition to HK-2 cells.

To further evaluate the *in vivo* toxicity of AA analogues, mice were administered single doses of AA I, AA II, AA IIIa, AA IVa, and AL I at 10, 20, and 40 mg/kg, respectively. The control group received 0.05% CMC-Na. Mice administered with AA I achieved a remarkable reduction of body weight compared to the control group. In addition, the body weight decreased with the increasing doses of AA I for both male and female mice (Supplementary Figure S3). Slight weight loss was also observed in male and female mice treated with AA II (40 mg/kg) during the first 10 days. However, the body weight of female mice increased after 10 days and there was no significant difference with the control group. Regarding AA IIIa, AA IVa, and AL I, no distinct changes in body weight were observed between the different dosages and the control group. Female mice treated with AA IVa (40 mg/kg) experienced a drastic weight loss on day 5, but the weight returned to the same level as in the other groups on day 7.

**FIGURE 3:**
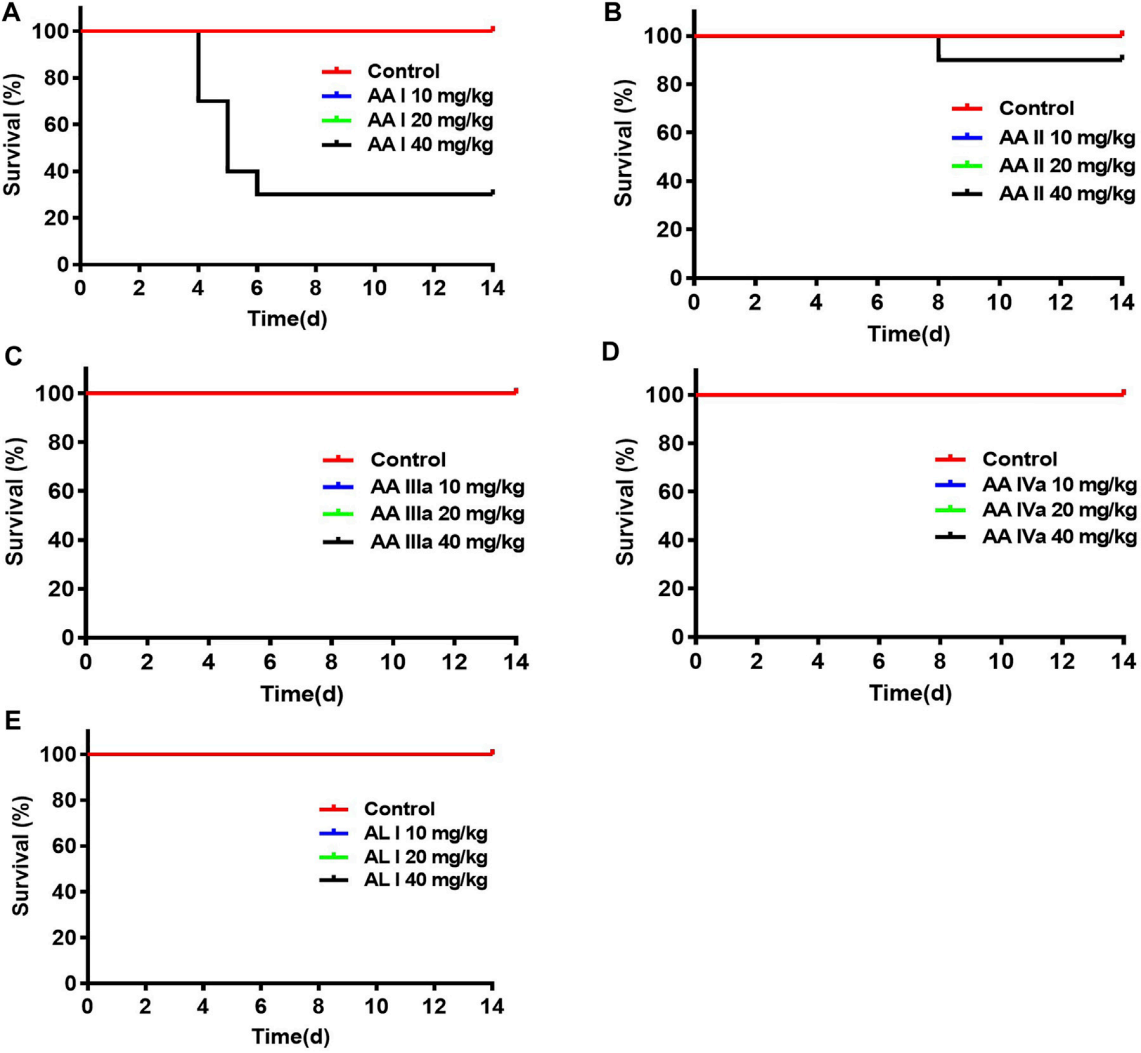
Survivals of mice administered with aristolochic acid analogues: AA I **(A)**, AA II **(B)**, AA IIIa **(C)**, AA IVa **(D)**, and AL I **(E)**. ICR mice were administered with aristolochic acid analogues at concentrations of 10, 20, and 40 mg/kg (*n* = 10 for each group). The curves were created by GraphPad Prism 7.0.

Survival of the control and treated groups were monitored for 14 days. As shown in Figure 3, mice (*n* = 7 of 10) treated with 40 mg/kg AA I were dead on days 4, 5, and 6 due to the acute kidney failure and the survival rate was 30%. 10% of mice died (*n* =1 of 10) on day 8 in the group treated with AA II (40 mg/kg). There was no death occurring in control, AA I (10, 20 mg/kg), AA II (10, 20 mg/kg), AA IIIa (10, 20, 40 mg/kg), AA IVa (10, 20, 40 mg/kg), AL I (10, 20, 40 mg/kg) treated groups.

**FIGURE 4:**
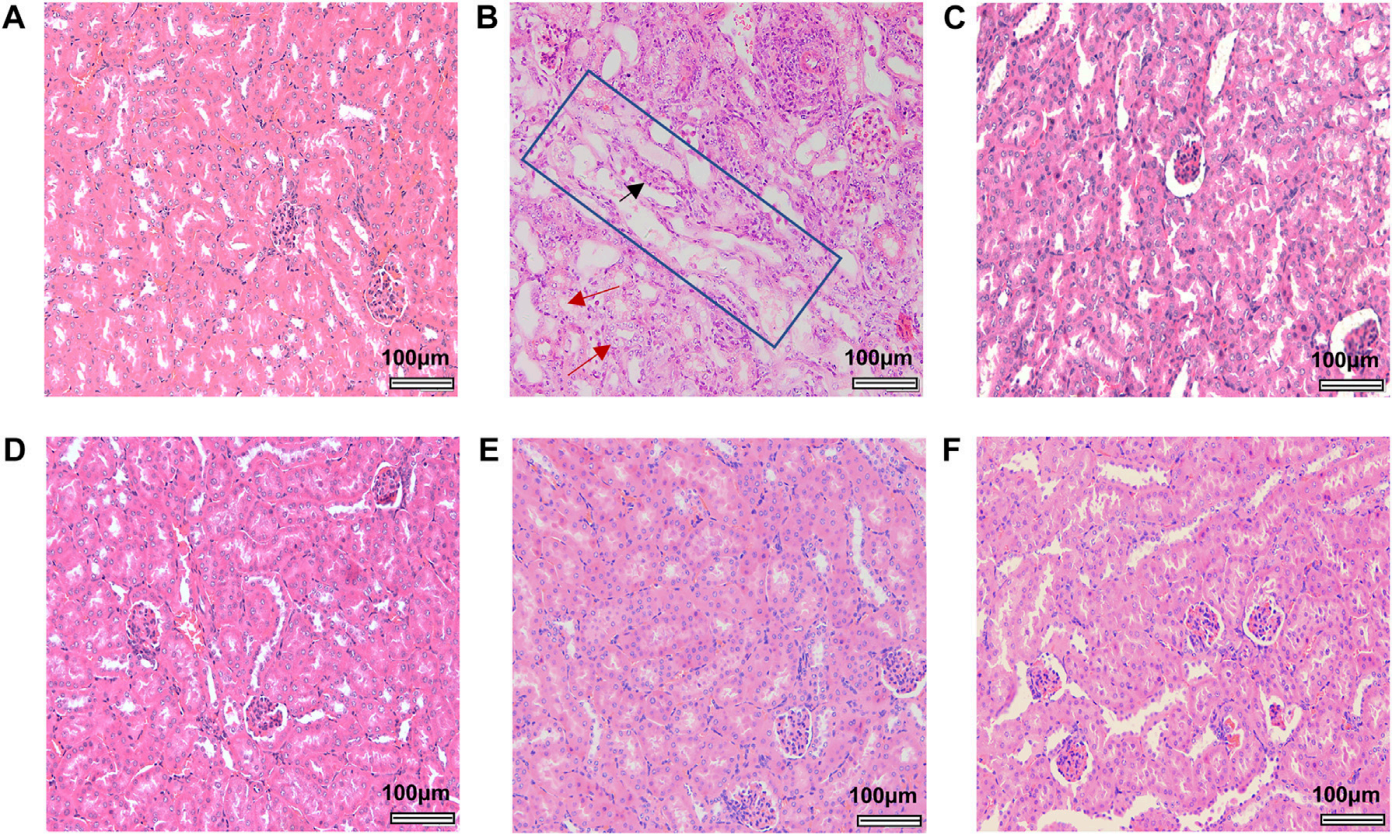
Histological images of renal tissues from control and aristolochic acid analogues treated male mice at a concentration of 40 mg/kg. Control **(A)**, AA I **(B)**, AA II **(C)**, AA IIIa **(D)**, AA IVa **(E)**, and AL I **(F)**. Large areas of renal tubule necrosis were outlined in the blue rectangle. Red arrows indicate areas of epithelial degeneration of renal tubules. Black arrow indicates the granular casts in renal tubule lumen. All the images were obtained at the same magnification: ×200, scale bar = 100 μm.

**FIGURE 5:**
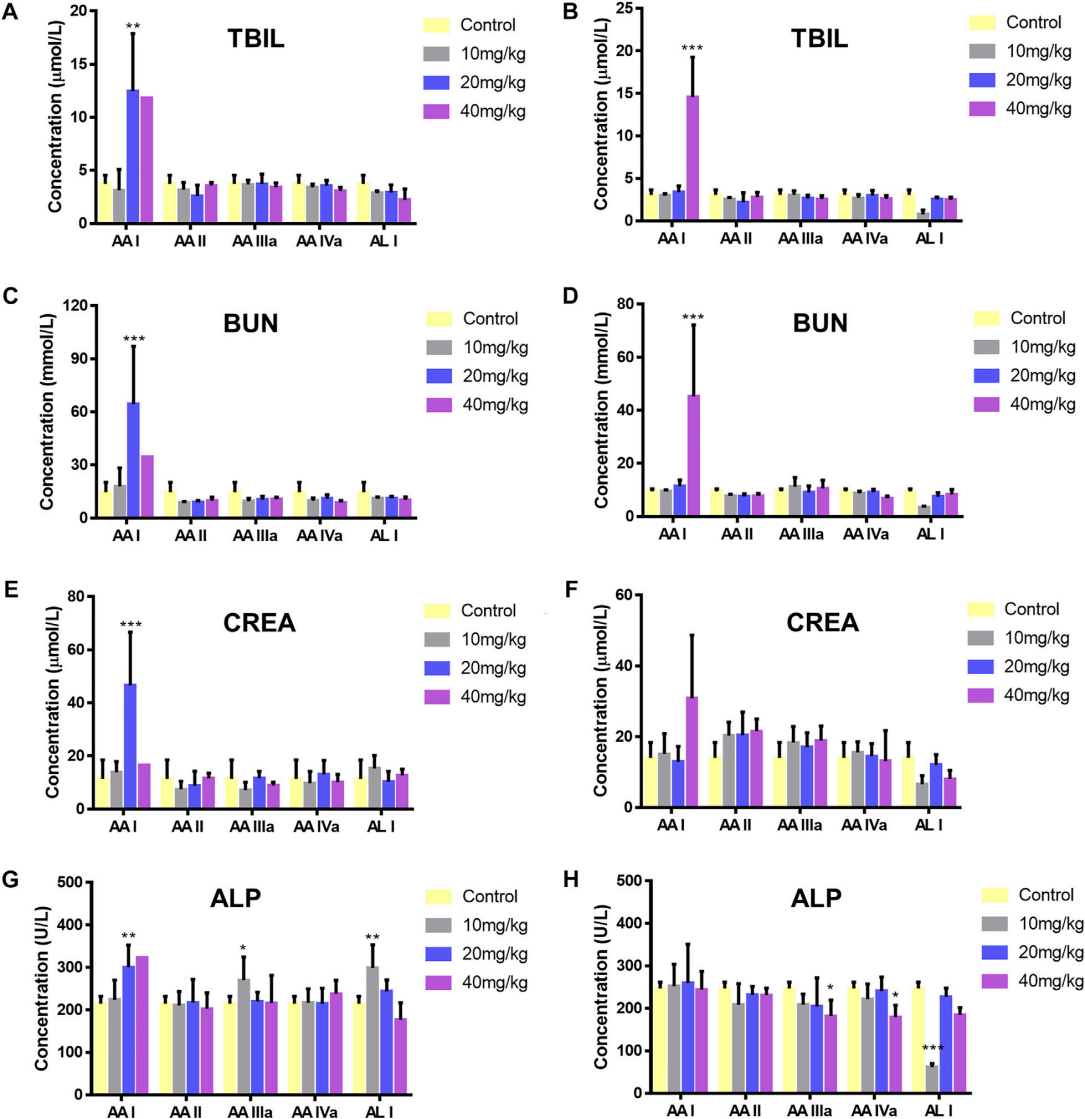
Level changes of TBIL (male, **(A)**; female, **(B)**), BUN (male, **(C)**; female, **(D)**), CREA (male **(E)**; female **(F)**), and ALP (male **(G)**; female **(H)**) of ICR mice administered with 0.5% CMC-Na, 10, 20, and 40 mg/kg aristolochic acid analogues. Significantly different from the control group (*p < 0.05; **p < 0.01, ***p < 0.001).

After mice were sacrificed, kidney and liver tissue were obtained for histopathological analysis. Significant lesions were only found in the renal tissues of mice treated with 40 mg/kg AA I. As shown in Figure 4, renal pathological changes were characterized with large area necrosis of renal tubules, epithelial degeneration of renal tubules, and granular casts in renal tubule lumen. Although there were different levels of changes in serum levels of TBIL, ALP, BUN, and CREA in AA I (20 mg/kg, 40 mg/kg) treated mice (Figure 5), no corresponding pathological changes were observed in the liver tissues of the same group (Supplementary Figure S5). The activities of AST, ALT, ALP and the concentrations of TBIL, TBA, CREA and BUN in AA II, AA IIIa, AA IVa, and AL I treated groups showed no significant difference compared to the control group, except for decreased AST, ALT, and ALP activity in the group treated with AL I (10 mg/kg). No pathological changes were observed in the liver tissues of mice treated with AL I.

### DNA Damages

**FIGURE 6:**
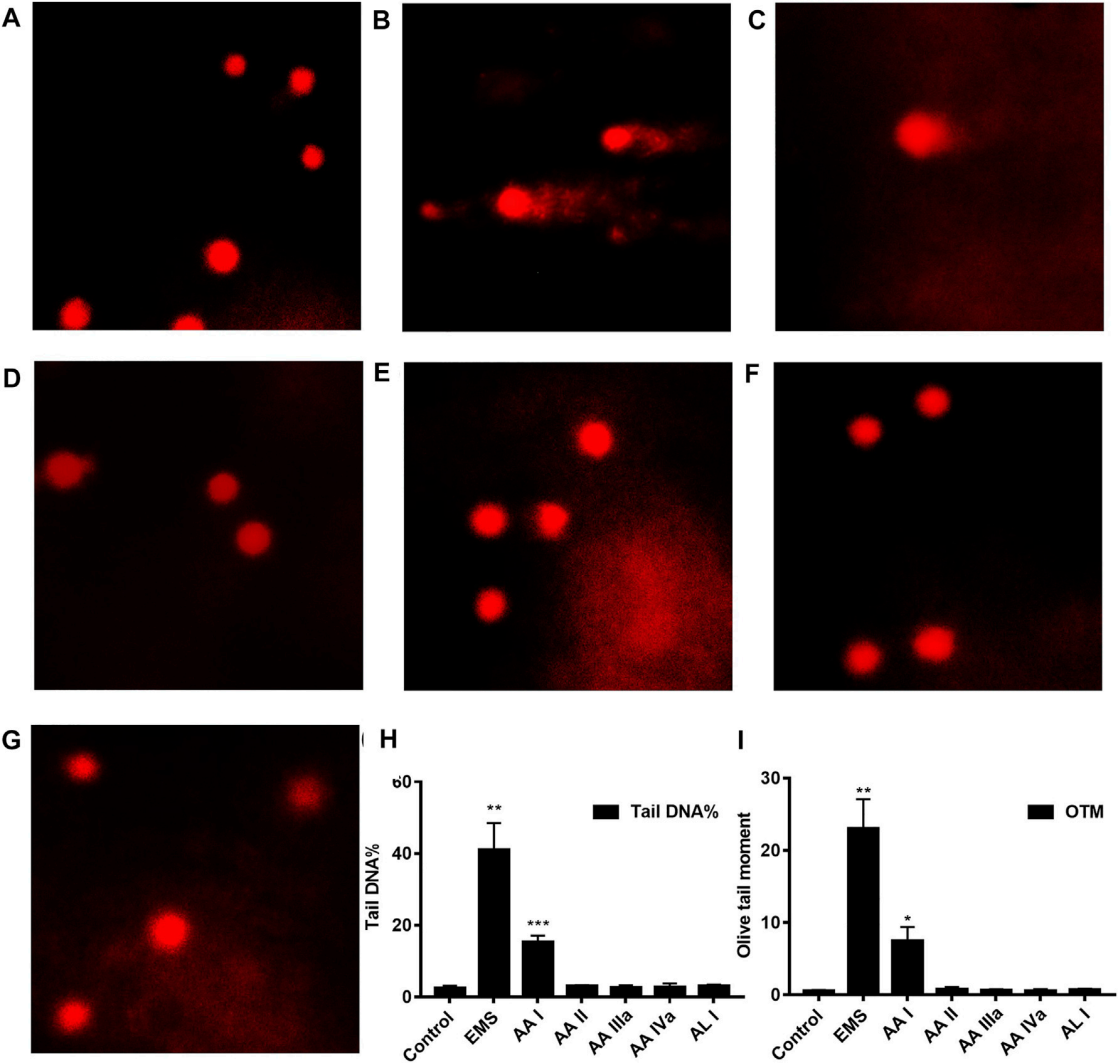
Comet assays: control **(A)**, EMS **(B)**, AA I **(C)**, AA II **(D)**, AA IIIa **(E)**, AA IVa **(F)**, AL I **(G)**. The tail DNA% **(H)** and olive tail moment (OTM) **(I)** of renal cells from mice treated with AA I (20 mg/kg). Data were calculated from 100 cells and one-way ANOVA was used for statistical and significance analysis. *p < 0.05; **p < 0.01; ***p < 0.001.

The comet assay is a simple and rapid method for detecting DNA damage in single cells. After cell lysis and DNA unwinding, undamaged DNA will remain in the nucleus and appear round during electrophoresis; however, cells with DNA damage resemble the shape of a comet as a result of the migration of negatively charged DNA from the nucleus toward the anode. The extent of DNA migration is positively correlated with the amount of DNA damage in cells (Speit and Rothfuss, 2012). We conducted comet assays to study the DNA damage of AA analogues. Ethyl methanesulfonate (EMS) was a strong genotoxic agent (Gocke et al., 2009) and was used as a positive control in this study. Hepatocyte and renal cells in the control group appeared round during electrophoresis, while EMS-treated hepatocyte and renal cells showed significant comet tailing (Figure 6, Supplementary Figure S6). Renal cells from AA I (20 mg/kg) treated mice showed an evident comet shape. The tail DNA% was 15.39 ± 1.75 (*p* < 0.001) and the olive tail moment was 7.45 ± 1.94 (*p* < 0.05), respectively (Supplementary Table S6). However, no DNA damage was observed in hepatocyte cells of mice treated with AA I (20 mg/kg). No DNA damage was observed in renal cells nor in hepatocyte cells in mice treated with AA II, AA IIIa, AA IVa, and AL I. These results indicated that AA I could cause direct DNA damage to the renal tissues, while the ability to induce DNA damage for AA II, AA IIIa, AA IVa, and AL I was relatively low at 20 mg/kg.

## Discussion

Plants of Aristolochiaceae have been gradually prohibited in the clinical use due to the nephrotoxicity and carcinogenicity of its component AA, with the exception of *Asarum* (Xixin), which has been used for centuries as an analgesic and antitussive (Wu et al., 2021). *Asarum* (Xixin) contains relatively low levels of AA I and AA II in roots and rhizomes; therefore, it is considered as a safe drug and is widely used in traditional Chinese medicines (Xie et al., 2007; Xue et al., 2008). In recent years, AA I was statistically associated with the incidence of hepatocellular carcinoma and the safety of the species of genus *Aristolochia* has once again aroused public concern (Hoang et al., 2013; Ng et al., 2017). Although the content of AA I in *Asarum* (Xixin) is required to be <0.001% according to the Chinese Pharmacopoeia 2020, the contents of AA analogues in herbs and their commercial products are still not known. In this study, the analogues of AA were quantitatively determined in 15 batches of *Asarum heterotropoides* F. Schmidt (Xixin) and 44 traditional Chinese patent medicines. AA I, AA II, and AA IIIa were not detected in *Asarum heterotropoides* F. Schmidt (Xixin) samples. Relative levels of AA IVa and AL I were found in all samples with species and regional differences, which was in consistent with previous studies (Hao et al., 2006; Zhang et al., 2008). Selecting the suitable species of *Asarum heterotropoides* F. Schmidt (Xixin) with less AA analogues content was an important way to improve the clinical safety of *Asarum heterotropoides* F. Schmidt (Xixin) and its formulated products. In this study, XX04 from Liaoning had some advantages on the low content of AA analogues. However, more samples of different species and origins need to be detected, in order to minimize the potential nephrotoxicity and hepatotoxicity.

Traditional Chinese patent medicines contain different types of medical constituents and the complex matrix brings difficulties in quantitative determination. The validation of a previous method is usually estimated based on the analysis of spiked samples of selected products in different forms and compositions, which makes the determination complicated (Vaclavik et al., 2014). In addition, the samples chosen were not representative of all samples. Herein, we established an effective and automatic solid phase extraction method for sample pretreatment, which improved the efficiency of sample processing and the accuracy of detection. In addition, selective enrichment of aristolochic acids and aristolactams can be achieved by adjusting the composition of the eluent. Aristolactams are easily eluted in neutral eluent, whereas AAs tended to be eluted in acidic mobile phase. The recovery of the method for the analogues of AA was within the range of 86.5–107.5% even at a concentration of 0.5–1.0 ng/ml. The method was successfully applied to the extraction of AA analogues and to the determination of traditional Chinese patent medicines. The AA analogues content was at *ng/g* in most samples. Although processing can reduce the content of *Aristolochia* AAs to varying degrees (Yang B. et al., 2012; Yuan et al., 2017), the contents of AA I in samples with processed *Aristolochia debilis* Siebold & Zucc. were still much higher than those with *Asarum* (Xixin). Thus, special attention should be paid to the potential nephrotoxicity or hepatotoxicity. The AA analogues varied in different dosage forms of the same prescription. Species and origins of *Asarum heterotropoides* F. Schmidt (Xixin) with different contents of AA analogues may be one of the reasons. The processing method also affected the content of active components, suggesting that optimizing processing may be another approach for reducing the potential toxicity.

AA I and AA II are considered as the main contents and causative agents of the nephrotoxic and carcinogenic effects of *Aristolochia* species (Debelle et al., 2008). However, in our study, AA IVa and AL I were found to be relatively high contents of *Asarum heterotropoides* F. Schmidt (Xixin) and its formulated products. Previous studies focused on the toxicity of AA I and AA II, while little is currently known about the toxicity of AA IIIa, AA IVa, and AL I, especially in terms of *in vivo* toxicity and carcinogenicity. To clarify whether these AA analogues contribute to the process of renal damage, both *in vitro* and *in vivo* toxicity assays were performed. Aristolochic acids I, II, IIIa, IVa, and aristolactam I showed varying degrees of cytotoxic effects to HK-2 cells, with AA-I and AL-I exhibiting the strongest cytotoxicity after 48 h of exposure. The localization of functional groups in the structure played a decisive role in cytotoxicity (Balachandran et al., 2005). AL I was the main reductive and genotoxicity metabolite of AA I. The relative cytotoxicity of AA I and AL I was contrary (Balachandran et al., 2005; Li J. et al., 2010). In this study, AA I showed higher cytotoxicity than AL I after 24 h, but the situation reversed after 48 h of incubation. This suggested there was a persistent cytotoxic effect of AL I, which was consistent with the long-term accumulation of AL I in the cytoplasm (Shang et al., 2008). Severe nephrotoxicity with large area necrosis was observed in mice treated with 40 mg/kg AA I. DNA damage was a major cause of nephrotoxicity, and this was confirmed by the comet shape of DNA in the AA I treated group. A high dosage of AA I can also induce hepatotoxicity with a significant increase in serum TBIL, BUN, and CREA levels and ALP and ALT activities. No obvious nephrotoxicity or hepatotoxicity was observed in AA II-, IIIa-, and IVa-treated mice in acute toxicity tests, which was consistent with the low cytotoxicity observed *in vitro*. Although the direct cytotoxicity of AL I was stronger than AA I, the induced DNA damage or nephrotoxicity of AL I was much lower than that of AA I. The weaker ability of AL I to induce mutagen, apoptosis, *TGFβ1*, and *FN* secretion may have played a role (Schmeiser et al., 1986; Li B. et al., 2004). As the metabolite of AA I and the substance that eventually binds to DNA, the toxicity of AL I, especially its long-term mutagenicity, still needs to be further studied, although no renal or DNA damage was observed in the acute toxicity test.

In conclusion, our study revealed that AA IVa and AL I were presented in relatively high levels in *Asarum heterotropoides* F. Schmidt (Xixin). Most traditional Chinese patent medicines in the study contained AA I, AA IVa, and AL I and the contents were at the *ng/g* level. Among the AA analogues studied, AA I exerted the strongest nephrotoxicity, while AA II, AA IIIa, and AA IVa showed weak toxicity both *in vitro* and *in vivo*. AL I showed significant cytotoxicity to HK-2 cells, but no renal toxicity was observed *in vivo* at 40 mg/kg. The contents of analogues of AA in *Asarum heterotropoides* F. Schmidt (Xixin) and its formulated products were far below the concentrations that induce acute nephrotoxicity; therefore, consumption of *Asarum heterotropoides* F. Schmidt (Xixin) with controlled doses and periods is relatively safe. However, considering the relatively high contents of AA IVa and AL I in *Asarum heterotropoides* F. Schmidt (Xixin), as well as the high cytotoxicity of AL I, the long-term toxicity of AA IVa and AL I should be further studied. In addition to the above-mentioned findings, the present study provides a basis for a broader quantitative determination of AA analogues and has a guiding implications for the safe clinical application of *Asarum heterotropoides* F. Schmidt (Xixin) and its formulated products.

## Data Availability

The original contributions presented in the study are included in the article/Supplementary Material; further inquiries can be directed to the corresponding author.

## Abbreviations

AAA, aristolochic acid analogues; AA I, aristolochic acid I; AA II, aristolochic acid II; AA IIIa, aristolochic acid IIIa; AA IVa, aristolochic acid IVa; AL I, aristolactam I; ALP, alkaline phosphatase activities; ALT, alanine amino transferase; AST, aspartate amino transferase; BEN, Balkan endemic nephropathy; BUN, blood urea nitrogen; CREA, creatinine; EMS, ethyl methanesulfonate; MRM, multiple reaction monitoring; SPE, solid phase extraction; TBA, total bile acid; TBIL, total bilirubin; UUC, upper tract urothelial carcinoma.
