# Supplementary material for: Quantitative Determination and Toxicity Evaluation of Aristolochic Acid Analogues in Asarum heterotropoides F. Schmidt (Xixin) and Traditional Chinese Patent Medicines
Source: Front Pharmacol. 2021 Nov 26;12:761593. doi: 10.3389/fphar.2021.761593 (PMC8662950; doi:10.3389/fphar.2021.761593)
Supplement: Supplementary file 1 [file DataSheet1.docx]

Supplementary Material

1. **Quantitative determination of aristolochic acid analogues (AAAs) in *Asarum heterotropoides* F. Schmidt (Xixin)**

Chromatographic separation of aristolochic acid analogues was obtained by gradient elution using the Agilent Eclipse XDB C18 column. The addition of formic acid (0.1%) greatly improved the separation performance. AAAs of AA I, AA II, AA IIIa, AA IVa and AL I were eluted at 24.36, 20.81, 9.36, 11.23 and 20.31 min, respectively. (**Supplementary Figure 1**).

An appropriate amount of AA I, AA II, AA IIIa, AA IVa, and AL I standards were dissolved separately with methanol for stock solutions. Mixed standards of AA I, AA II, AA IIIa, AA IVa and AL I at concentrations of 115.2, 76.0, 85.6, 172.6 and 35.2 μg/mL were then progressively diluted to examine the linearity of HPLC analysis. Calibration curves were prepared at seven different concentrations, including the lower limit of quantification.

**Supplementary Figure 1**. Chromatograms of aristolochic acid analogues in standard solutions and *Asarum heterotropoides* F. Schmidt (Xixin, XX01).

The intra-day and the inter-day precisions were obtained by analyzing five aliquots of each quality control (QC) sample on the same day and the QC samples on three different days. Results showed that the intra-day RSD values of AA I, AA II, AA IIIa, AA IVa and AL I were 0.75%, 2.2%, 0.38%, 0.47%, and 1.52%, respectively. And the inter-day RSD values were 1.55%, 4.02%, 1.50%, 4.61%, and 7.75%, respectively, indicating good repeatability of the instrument (**Supplementary Table 1**). The limit of detection (LOD) was defined as S/N≥ 3, while the lowest concentration of curve was targeted as LOQ.

The recovery of the method was assessed based on analysis of six replicate samples. Appropriate amounts of AAAs were added into the samples and then processed by HPLC. The recoveries of AA I, AA II, AA IIIa, AA IVa, and AL I were in the range of 97.55–113.27%. The stability of the samples (n=5) was tested at room temperature (25°C) for 72 h and the relative standard deviation (RSD) of AA I, AA II, AA IIIa, AA IVa and AL I was below 7.99%.

**Supplementary Table 1**. The linearity, precision and recovery of aristolochic acid analogues.

|  | **Range (μg/mL)** | **R^2^** | **LOD**  **(μg/mL)** | **LOQ**  **(μg/ mL)** | **RSD of Precisions (%)** | | **Recovery**  **(%)** |
| --- | --- | --- | --- | --- | --- | --- | --- |
|  |  |  |  |  | **Intra-day** | **Inter-day** |  |
| AA I | 3.6-115.2 | 0.9999 | 0.09 | 0.33 | 0.75 | 1.55 | 107.65±2.28 |
| AA II | 2.4-76.0 | 0.9999 | 0.09 | 0.28 | 2.2 | 4.02 | 97.55±3.19 |
| AA IIIa | 2.6-85.6 | 0.9998 | 0.03 | 0.09 | 0.38 | 1.50 | 104.45±2.30 |
| AA IVa | 5.3-172.6 | 0.9999 | 0.07 | 0.23 | 0.47 | 4.61 | 113.27±2.61 |
| AL I | 1.1-35.2 | 0.9999 | 0.07 | 0.23 | 1.52 | 7.75 | 104.83±5.87 |

1. **Quantitative determination of aristolochic acid analogues in commercially available traditional Chinese patent medicines (TCM)**

For quantitative analysis, the [M+NH_4_]^+^ ions were used as the precursor ions of AA I, AA II, AA IIIa, and AA IVa, while [M+H]^+^ ion was detected as the base peak of AL I. [M+H-CO_2_]^+^ ions were used as the MS/MS product ions of AA I and AA II. [M+H-NO_2_]^+^ ions were used as the MS/MS product ions of AA IIIa, and AA IVa. As for AL I, [M+H-CH_3_]^+^ ion was the main MS/MS product ion. The cone voltage and collision energy for each aristolochic acid analogue was listed in **Supplementary Table 2**. The aristolochic acid analogues were well separated by UPLC and the retention time of AA I, AA II, AA IIIa, AA IVa and AL I were 6.17, 5.77, 4.42, 5.10, 7.09 min, respectively (**Supplementary Figure 2**).

Appropriate amounts of standards were dissolved separately in methanol at concentrations of 50 or 100 μg/mL. All solutions were stored in the refrigerator at -20ºC before usage. Calibration curves were obtained by analysis of eight different concentrations of mixed standards of aristolochic acid analogues in this study. The linear ranges were 0.50–200 ng/mL for AA I, AA II, AA IIIa, AA IVa and 0.25-100 ng/mL for AL I. The linear equations are shown in **Supplementary Table 3**. The calibration curves showed good linearity for each analyte with R^2^ ≥ 0.9978.

**Supplementary Table 2.** MRM parameters for quantitative determination of aristolochic acid analogues.

| **Compounds** | **Parent (m/z)** | **Daughter(m/z)** | **Cone (V)** | **Collision (eV)** |
| --- | --- | --- | --- | --- |
| AA I | 359.1 | 298.1 | 38 | 10 |
| AA II | 329.1 | 268.1 | 16 | 10 |
| AA IIIa | 345.2 | 282.0 | 35 | 13 |
| AA IVa | 375.0 | 312.0 | 20 | 13 |
| AL I | 294.0 | 279.0 | 52 | 28 |

**
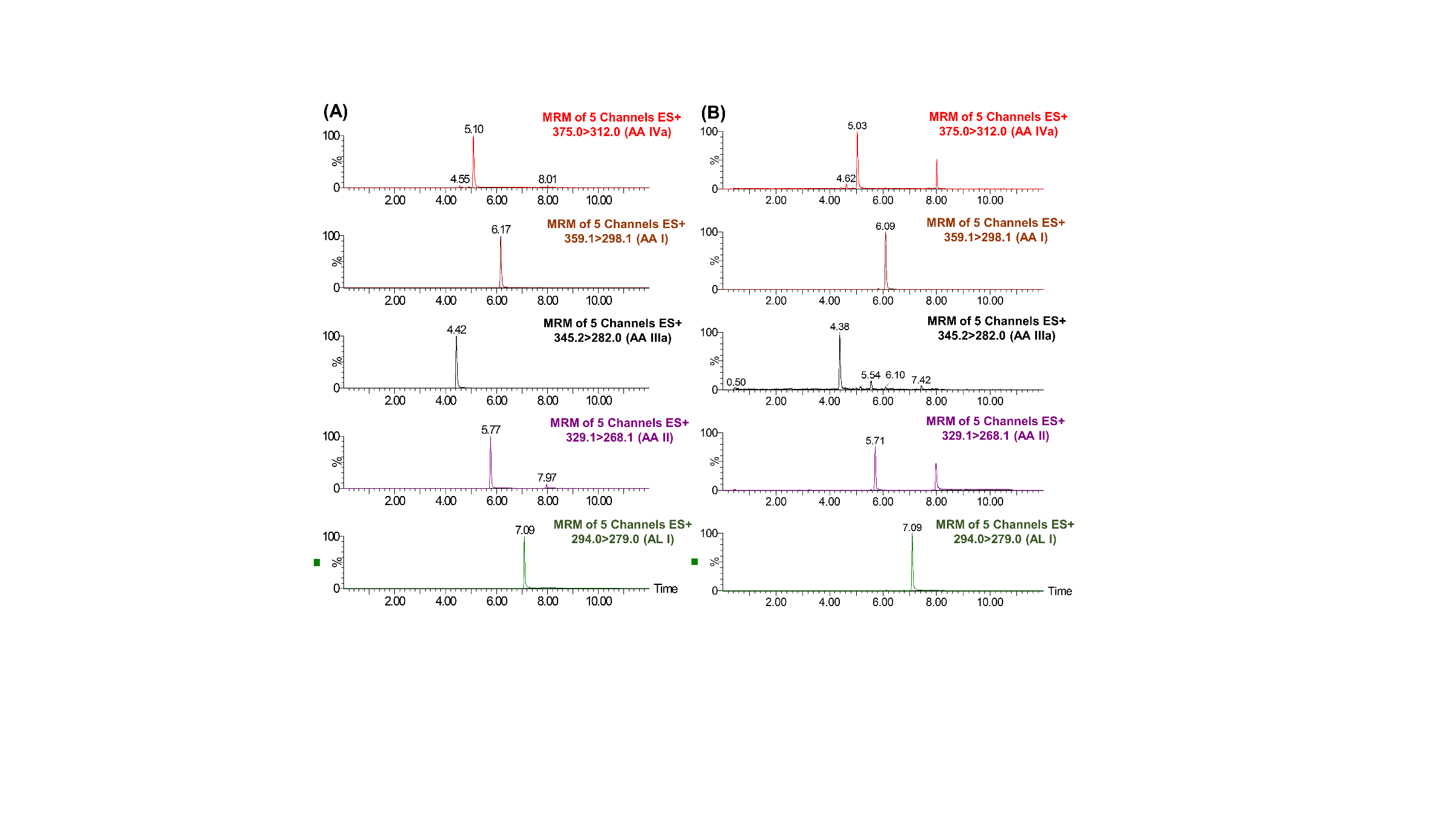
**

**Supplementary Figure 2**. Representative multiple reaction monitoring chromatograms of analytes in positive ion mode. (A) QC samples of aristolochic acid analogues. (B) Chromatograms of aristolochic acid analogues detected in the Duzhongzhuanggu capsule samples.

**Supplementary Table 3.** The linearity of UPLC-MS/MS method for the quantitative detection of aristolochic acid analogues.

| **Compounds** | **Range (ng/mL)** | **Linear equation** | **LOD**  **(ng/mL)** | **LOQ**  **(ng/mL)** | **R^2^** |
| --- | --- | --- | --- | --- | --- |
| AA I | 0.50-200 | y=6456.47x+2023.17 | 0.01 | 0.50 | 0.9994 |
| AA II | 0.50-200 | y=2419.22x+235.716 | 0.03 | 0.50 | 0.9999 |
| AA IIIa | 0.50-200 | y=1124.73x+12.197 | 0.03 | 0.50 | 0.9995 |
| AA IVa | 0.50-200 | y=809.235x+82.7487 | 0.04 | 0.50 | 0.9990 |
| AL I | 0.25-100 | y=12420.1x+574.486 | 0.01 | 0.25 | 0.9978 |

The accuracy, inter-day and intra-day precisions were assessed by analysis of six aliquots of QC samples at three different levels (1, 40, 160 ng/mL for AA I, AA II, AA IIIa, AA IVa and 0.5, 20, 80 ng/mL for AL I). The accuracy was ranged from 85.89% to 113.35%, which met the recommended limits (85–115%). The intra-day and inter-day precision were below 10% and 8% (RSD), respectively (**Supplementary** **Table 4**).

The LOD was defined as S/N ≥ 3, while the LOQ was the lowest concentration on the curve. The LOD and LOQ values of 5 analytes were shown in **Supplementary Table 3.**

The recovery of the method was validated by analysis of three levels of QC samples which were processed by established solid phase extraction. Results showed the recovery of aristolochic acid analogues was in the range of 86.50–111.88%. Stability of QC samples (n=6) was tested at 12°C for 48 h. The RSD values of analytes were between 0.94–4.64%, which suggested a good sample stability at temperature 12 °C for 48 h.

**Supplementary** **Table 4.** The accuracy, precision and recovery of aristolochic acid analogues.

| Compounds | Conc. (ng/mL) | Accuracy and Precision | | | | Recovery (%) |
| --- | --- | --- | --- | --- | --- | --- |
|  |  | Intra-day | RSD (%) | Inter-day | RSD (%) |  |
| AA I | 1 | 0.97±0.05 | 5.34 | 0.99±0.07 | 6.81 | 86.50±5.00 |
|  | 40 | 39.17±0.40 | 1.03 | 41.4±2.14 | 5.22 | 106.69±3.40 |
|  | 160 | 150.30±3.36 | 2.24 | 155.42±9.33 | 6.00 | 101.91±3.07 |
| AA II | 1 | 0.98±0.04 | 4.15 | 1.00±0.00 | 0.00 | 97.50±5.00 |
|  | 40 | 41.12±0.54 | 1.32 | 40.97±1.08 | 2.63 | 111.88±1.20 |
|  | 160 | 164.87±0.98 | 0.59 | 166.52±1.34 | 0.81 | 107.73±3.66 |
| AA IIIa | 1 | 1.03±0.05 | 5.00 | 1.00±0.02 | 1.83 | 92.50±5.00 |
|  | 40 | 42.62±0.99 | 2.33 | 41.03±1.18 | 2.88 | 107.00±1.85 |
|  | 160 | 168.58±1.92 | 1.14 | 161.95±5.46 | 3.37 | 107.34±1.84 |
| AA IVa | 1 | 0.98±0.08 | 7.66 | 1.01±0.07 | 7.28 | 87.50±5.00 |
|  | 40 | 42.45±0.60 | 1.41 | 41.07±1.32 | 3.21 | 96.56±1.64 |
|  | 160 | 152.94±1.78 | 1.73 | 157.97±7.53 | 4.77 | 98.86±4.35 |
| AL I | 0.5 | 0.55±0.05 | 9.96 | 0.56±0.02 | 3.88 | 107.50±5.00 |
|  | 20 | 18.75±0.31 | 1.64 | 19.38±0.66 | 3.41 | 99.00±2.48 |
|  | 80 | 83.12±1.62 | 1.94 | 83.66±0.99 | 1.18 | 104.16±1.86 |

Traditional Chinese patent medicines containing ingredient known to contain aristolochic acids or likely to be substituted for an *Aristolochia spp.* were extracted and determined by the method established. Each sample was prepared with four parallels. After extraction, the samples were detected immediately by UPLC-MS/MS. The contents of AA I, AA II, AA IIIa, AA IVa, and AL I are listed in **Supplementary Table 5.**

**Supplementary Table 5.** Quantitative determination of aristolochic acid analogues in traditional Chinese patent medicines.

| **Product number** | **Samples** | **Form** | **Ingredient known to contain AA or likely to be substituted for an *Aristolochia spp.*** | **Contents (μg/g)^a^** | | | | |
| --- | --- | --- | --- | --- | --- | --- | --- | --- |
|  |  |  |  | **AA I** | **AA II** | **AA IIIa** | **AA IVa** | **AL I** |
| 1 | Guxian | Tablet | *Stephania tetrandra* S.Moore (Fangji) | 0.020 ± 0.008 | ND | ND | 0.016 ± 0.003 | 0.021 ± 0.005 |
| 2 | Fuming | Tablet | *Akebia quinata* (Houtt.) Decne*.* (Mutong) | 0.017 ± 0.007 | ND | ND | 0.024 ± 0.008 | 0.016± 0.006 |
| 3 | Ershiwuweisongshi | Watered pill | *Aristolochia contorta* Bunge. (Madouling) | 52.450 ± 2.715 | 0.223 ± 0.017 | 0.135 ± 0.015 | 0.440 ± 0.039 | 0.863 ±0.0 46 |
| 4 | Qingfeizhike | Watered pill | *Aristolochia contorta* Bunge. (Madouling) | NQ | ND | NQ | ND | 0.015 ±0.002 |
| 5 | Shenqijianwei | Granule | *Inula helenium* L. (Tumuxiang) | ND | ND | ND | ND | ND |
| 6 | Xiaofengzhiyang | Granule | *Akebia quinata* (Houtt.) Decne*.* (Mutong) | 0.015 ± 0.002 | ND | 0.010 ± 0.001 | 0.014 ± 0.002 | 0.018 ± 0.002 |
| 7 | Shenmeiyangwei | Granule | *Aristolochia debilis* Siebold & Zucc. (Qingmuxiang) | ND | ND | ND | 0.016 ± 0.002 | ND |
| 8 | Guanxisuhe | Capsule | *Aristolochia debilis* Siebold & Zucc. (Qingmuxiang) | ND | ND | ND | ND | 0.008 ± 0.001 |
| 9 | Yangweining | Capsule | *Inula helenium L.* (Tumuxiang) | ND | ND | ND | ND | 0.018 ± 0.001 |
| 10 | Jingzhikesoutanchuan | Watered pill | *Aristolochia debilis* Sieb. et Zucc. (Madouling), *Asarum heterotropoides* F. Schmidt (Xixin) | 3.416 ±0.086 | 0.301 ± 0.018 | 1.234 ± 0.049 | 1.287 ± 0.067 | 3.242 ±0. 137 |
| 11 | Duzhongguanggu | Capsule | *Asarum heterotropoides* F. Schmidt (Xixin), *Aristolochiae mollissimae* Hance (Xungufeng) | 4.110 ± 0.102 | 0.210 ± 0.056 | 0.290 ± 0.036 | 0.780 ± 0.026 | 1.193 ± 0.020 |

Note: All the data were shown as mean ± SD, and the mean value is obtained by averaging four parallel samples. “ND” not detected; “NQ” detected, but below the limit of quantitation. ^a^, μg/g means μg (AAAs)/g (traditional Chinese patent medicines)

1. ***In vivo* toxicity assays**

**Supplementary Figure 3.** Body weight changes of ICR mice after single administration with 0.5% CMC-Na, 10, 20, 40 mg/kg aristolochic acid analogues. Mice were observed for 14 days and the body weight was recorded every two days. AA I, male (**A**); AA I, female (**B**); AA II, male (**C**); AA II, female (**D**); AA IIIa, male (**E**); AA IIIa, female (**F**); AA IVa, male (**G**); AA IVa, female (**H**); AL I, male (**I**); AL I, female (**J**).

**Supplementary** **Figure 4**. Level changes of AST (male, A; female, B), ALT (male, C; female, D), TBA (male E; female F) of ICR mice administrated with 0.5% CMC-Na, 10, 20, 40 mg/kg aristolochic acid analogues. Significantly different from the control group (^*^, *p* < 0.05; ^**^, *p* < 0.01, ^***^, *p* < 0.001).


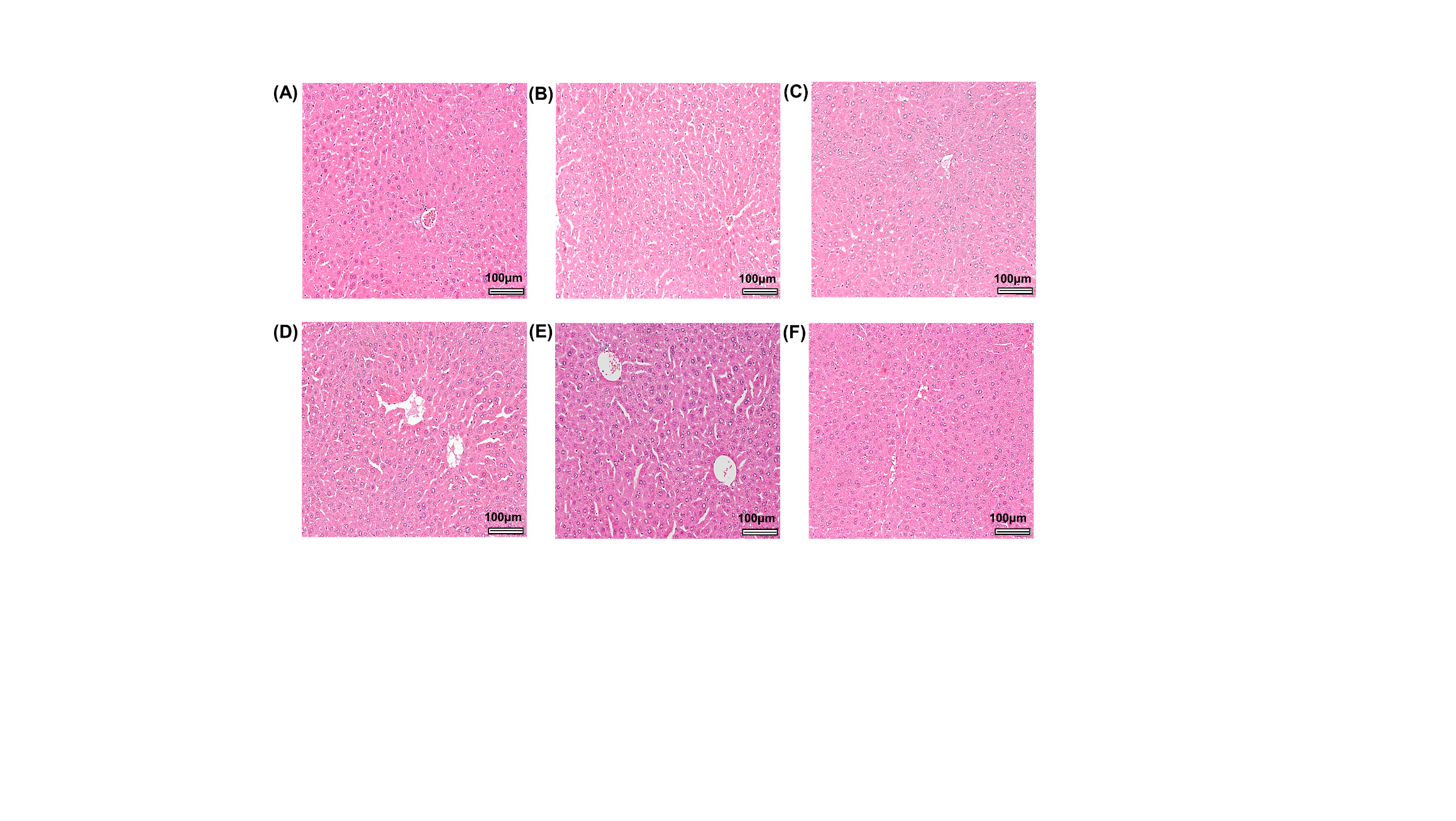


**Supplementary Figure 5**. Histological images of liver tissues from control and aristolochic acid analogues treated male mice at concentration of 40 mg/kg. Control (**A**), AA I (**B**), AA II (**C**), AA IIIa (**D**), AA IVa (**E**), AL I (**F**). All the images were obtained at the same magnification: ×200, scale bar = 100 μm.

1. **Comet assay**

Mice (n=35) were randomly divided into seven groups, with 5 mice in each group. Then they were administered with normal saline (NS), ethyl methanesulfonate (EMS) (577.6 mg/kg), and aristolochic acid analogues (20.0 mg/kg) for three days, respectively. After the final administration, mice were perfused and euthanized to obtain the renal and liver tissues.

The removed renal and liver tissues were minced into pieces and dispersed with 10 mL DMEM high-sugar culture medium. The mixture was filtered through a 200-mesh sieve and centrifuged at 800 rpm for 5 min. The supernatant was discarded and the cells were resuspended and centrifuged again. Then the cells were cultured in DMEM containing 10% FBS.

25 μL single-cell suspension (5000 cells) with 75 μL of 0.7% low melting point agarose gel were mixed and 100 μL of the mixture was dropped on the bottom layer of agarose gel. The prepared glass slides were stored in the refrigerator at 4℃ for 4 min, and then dipped into the alkaline cell lysates for 2 h at 4℃. Slides were removed and rinsed with PBS buffer for 3 times.

The alkaline electrophoresis buffer (pH 13.0) precooled at 4℃ was put into the electrophoresis tank. The tank was covered to avoid light and remained static at 4℃ for 20 min. The voltage was set at 0.8 V/cm, and the current was 300 mA during the electrophoreses.

After 20 min, the slides were removed and the excess salt was washed away. The slides were washed by 0.4 mol/L precooled Tris-HCl buffer for 5 min and then dehydrated by ethanol for 15 min. Then the slides were stained with 30 μL PI staining solution (2 μg/mL) in the dark for 15 s, and washed in water several times.

A fluorescence microscope was used for visualization of DNA damage. A total of 100 randomly selected cells from kidney or liver tissues were analyzed. Parameters such as tail DNA percentage and Olive Tail Moment were calculated by the Comet Assay Software Project (CASP, V1.2.3).

**Supplementary Table 6.** The tail DNA percentage (tail DNA %) and the Olive Tail Moment of single cell suspensions in kidney and liver tissues of mice after exposure to EMS or aristolochic acid analogues.

| **Groups** | **Dose (mg/kg)** | **Kidney** | | **Liver** | |
| --- | --- | --- | --- | --- | --- |
|  |  | **Tail DNA%** | **Olive Tail Moment (μm)** | **Tail DNA%** | **Olive Tail Moment (μm)** |
| Control | NS | 2.51±0.60 | 2.33±0.99 | 2.47±0.81 | 2.67±0.61 |
| EMS | 577.6 | 40.97±7.55^**^ | 23.01±4.09^**^ | 55.18±6.23^**^ | 33.60±6.11^**^ |
| AA I | 20.0 | 15.39±1.75^***^ | 7.45±1.94^*^ | 2.92±0.54 | 0.88±0.14 |
| AA II | 20.0 | 3.08±0.25 | 0.78±0.30 | 3.37±0.42 | 0.89±0.22 |
| AA IIIa | 20.0 | 2.60±0.69 | 0.61±0.16 | 3.09±0.47 | 0.92±0.20 |
| AA IVa | 20.0 | 2.80±0.99 | 0.57±0.22 | 2.98±0.22 | 0.81±0.14 |
| AL I | 20.0 | 3.12±0.33 | 0.66±0.21 | 3.26±0.52 | 0.74±0.29 |

Note: Number of animals: 5/group; Compared with control group: ^*^, *p* < 0.05; ^**^, *p* < 0.01; ^***^, *p* < 0.001.


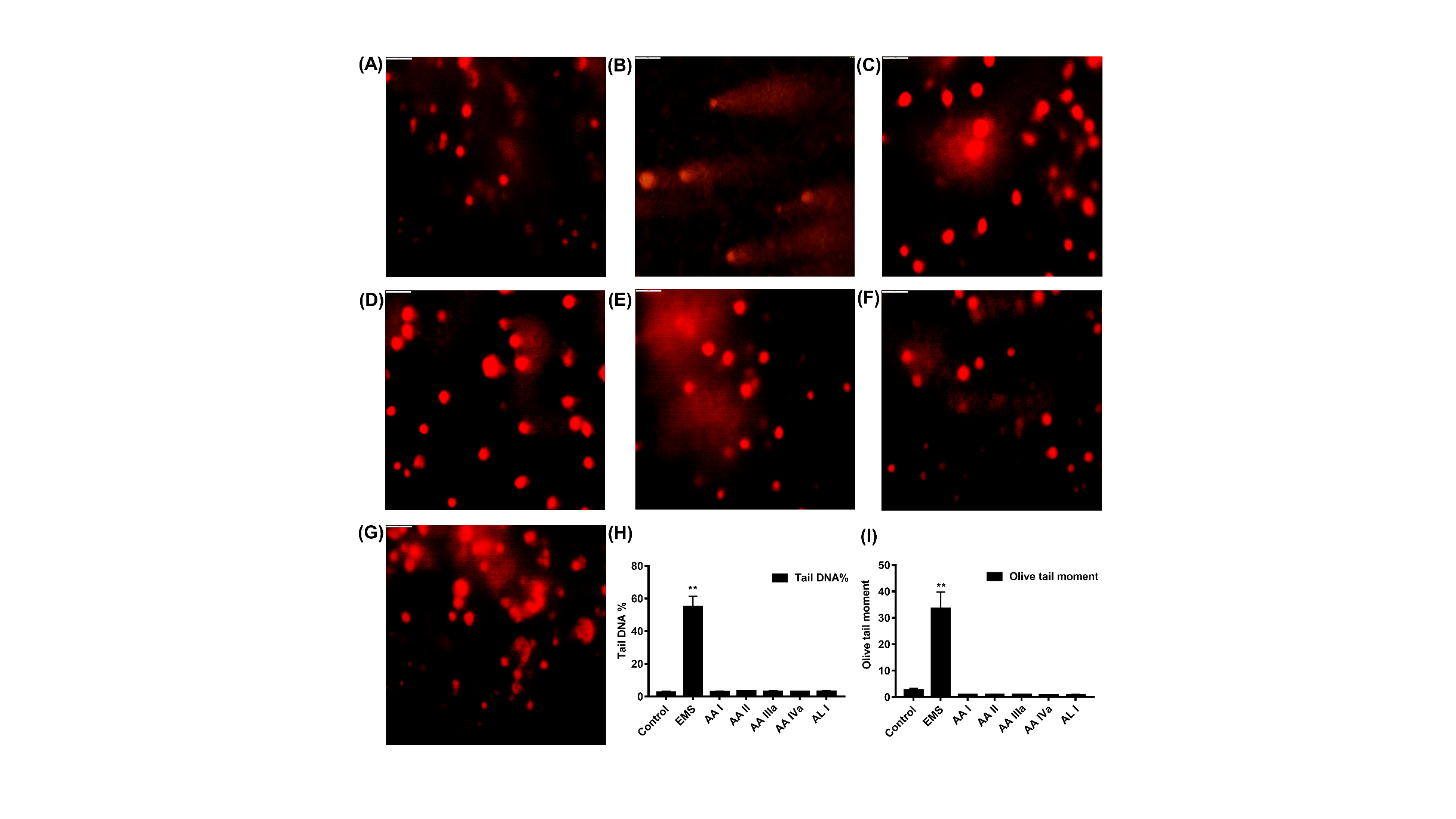


**Supplementary Figure 6**. Comet assays: Control (**A**), EMS (**B**), AA I (**C**), AA II (**D**), AA IIIa (**E**), AA IVa (**F**), AL I (**G**). The tail DNA% (**H**) and olive tail moment (**I**) of liver cells. Data were calculated from 100 cells and one-way analysis of variance (ANOVA) followed by least significance difference test were used for statistical and significance analysis. ^*^, *p* < 0.05; ^**^, *p* < 0.01; ^***^, *p* < 0.001.
